# Flattening Hierarchical Structures to Empower Women Trainee Leaders on Social Media Teams

**DOI:** 10.2196/47800

**Published:** 2023-06-05

**Authors:** Marah N Kays, Deborah D Rupert, Olivia Negris, Beatrix Thompson, Marla L Clayman, Lisa Mordell, Tricia Pendergrast, Eve Bloomgarden, Rakhee K Bhayani, Shikha Jain

**Affiliations:** 1 Kansas City University Kansas City, MO United States; 2 School of Medicine Stony Brook University Stony Brook, NY United States; 3 School of Medicine Rush Medical College Rush University Medical Center Chicago, IL United States; 4 School of Medicine Harvard University Boston, MA United States; 5 Center for Healthcare Organization and Implementation Research Department of Veterans Affairs Bedford, MA United States; 6 Stritch School of Medicine Loyola University Chicago Maywood, IL United States; 7 Northwestern University Feinberg School of Medicine Chicago, IL United States; 8 NorthShore University Health System Chicago, IL United States; 9 Department of Medicine Washington University School of Medicine St. Louis, MO United States; 10 Division of Hematology and Oncology University of Illinois at Chicago Chicago, IL United States

**Keywords:** social media, medical education, gender equity, women, empowerment, pyramidal hierarchy, residency, medical training, health care, women empowerment

## Abstract

We share our experience empowering women trainees and leadership through a flattened hierarchical social media team structure with supporting evidence from measurable outcomes.

Traditional academic medical settings hold to pyramidal hierarchies in which faculty oversee the training of resident physicians and medical students (“trainees”), with a one-way mentorship model [1]. The rigidity of this structure often fails to engage trainees in ways that optimize their meaningful contributions, resulting in the underuse of their unique skill sets [2,3]. The traditional mentorship model contributes to damaging outcomes including the jeopardization of trainee autonomy [4,5]. For example, trainees’ inherent lack of power is associated with hesitancy to speak out against harassment or when witnessing medical errors [4,6,7]. The consequences of the traditional mentorship model are likely to be particularly pronounced for women and underrepresented trainees who are more hesitant to “overstep” in formal academic climates [8,9].

Matriculating medical trainees represent increasingly diverse demographic backgrounds (gender, age, race/ethnicity) [10,11]. Trainee diversity adds unique perspectives and expertise, as well as a heightened commitment to policy and advocacy work [12]. To forgo fully engaging the voices of the next generation of future physicians is a substantial loss to academic culture. We suggest the benefits of deformalizing mentorship and promoting more equitable, bidirectional interactions include more innovative strategies for success in our health care systems and improved recruitment and retention [13]. However, the direct impact of a more dynamic system that better engages trainees from vulnerable populations (women, underrepresented minority or historically marginalized, LGBTQ+ [lesbian, gay, bisexual, transgender, queer], etc, communities) is often challenging to measure.

Social media platforms are examples of systems that inherently deconstruct traditional academic hierarchies. This makes social media a particularly apt means of examining bidirectional mentorship and trainee empowerment. Additionally, social media engages and represents a largely untapped trainee skill set; one that lies at the intersection of science communication and technological savviness [14]. Those areas of expertise are increasingly relevant to health care professionals and their careers [15]. However, senior physicians typically lack training and expertise in content curation, leaving them unequipped to engage on social media platforms [16]. Another attractive feature for using social media to test a nonhierarchical model is publicly blinded leadership where a trainee’s title or ranking within academia need not dictate the perceived quality of the content.

Here, we describe a case study in which the social media skill sets and expertise of women trainees were harnessed to flatten the committee structure within a medical advocacy organization. We implemented a bidirectional mentorship model, a new dynamic step beyond the previously studied “reverse mentorship,” to the existing social media committee of our advocacy-focused nonprofit organization within the health care space, Women in Medicine (WIM)—a 501(c)(3) organization that empowers women health care workers [13]. The goals of the social media committee within that organization have been: first, to reach their target audience and grow the platform’s “followings”; second, to retain women trainee leadership, team membership, and structure; and third, to impart social media skills to senior academics looking to acquire these capabilities.

The WIM social media committee was composed of self-selected medical academics of various ranks (n=15). Each committee member’s role was determined from their self-reported prior experience and relevant skill sets. Particular emphasis was placed on graphic design software, website building and management, advertisement, and public relations experience. Women trainees with extensive existing skill sets were named as team coleads, while faculty provided expertise and oversight on public-facing medical content. Similar to previous work, we found that this structure adequately harnessed the expertise of both our trainee and senior physician populations [17]. At the onset of restructuring, the committee was asked to outline and implement a strategy using the SMART (specific, measurable, achievable, relevant, and time-bound) goals approach that detailed means for achieving its goals. This strategy included increasing engagement and disseminating content using infographics, strategic community partnerships, and targeted hashtag campaigns.

We examined the success of the social media committee following hierarchical flattening and the instatement of women trainee leadership compared to when trainees were part of the social media committee but not in leadership positions. Prior to the trainee leadership installment, the organizations had an established social media committee and social media presence (approximately 1500 followers on their Twitter platforms). The WIMS Twitter growth rate increased 1.7 times compared to the year prior, in which leadership was not positioned among trainees. Again, simple linear regressions of the follower growth rate in the year following compared to the year prior suggested that trainee leadership significantly improved the reach of the account (simple linear regression *R*^2^=0.97 and *R*^2^=0.97, respectively; *P*<.001). Additionally, on average, there was a 2.7-fold increase in the number of monthly impression tweets received compared to the year prior in which leadership was not positioned among trainees (1-tailed, unpaired *t* test *P*=.02). The most successful single campaign produced over 400,000 impressions related to the WIM conference, more than five times the previous WIM record. Moreover, the WIM account was officially verified by the Twitter platform—previously considered a symbol of “authentic, notable, and active” accounts per Twitter’s description prior to the recent acquisition of that platform. The most recent trends in our data substantiate the continued success of the organization (and of trainee leaders) on these social media platforms; in the 2 years following instatement of trainee leadership, followership growth rates remained consistent, gaining followers at a statistically equivalent rate as in the first trainee-lead year (*R*^2^=0.97 and *R*^2^=0.94, respectively; *P*=.15, not significant) [18].

We hold that these short- and long-term objective outcome measures strongly argue for increased overall effectiveness of the restructured social media community, including more successful science communication, public engagement, and leadership. Subjective *outcomes* also support the success of our model. Faculty from the social media team reported learning new social media skills that they have implemented for their own social media brands including accounts that represent individuals and institutional programs (eg, graduate medical education program or departmental handles). A trainee lead continues to direct the committee and all committee members have remained in place from the time of restructuring, suggesting their buy-in to this model.

The bidirectional mentorship exemplified here suggests that reimagined academic team structures can improve the achievement of specific, metric-driven goals; facilitate open discussion among teams; empower trainee leaders, especially those with historically marginalized voices [19]; promote the learning of unique professional skill sets among team members; and provide a “learners as teachers” opportunity.

The next generation of leaders in medicine brings unique perspectives to the health care milieu. We hold that trainees, and their skill sets, are better engaged when medical hierarchical structures are softened. Here we have reflected on our own experience empowering women trainee leadership with a bidirectional model of mentorship and recounted experiences from an advocacy-focused team wherein women trainees’ skills have greatly facilitated the growth of public health and gender equity advocacy efforts on social media. We encourage teams with mixed faculty and trainee makeup to consider where and how they might diversify teaching and leadership roles and the potential benefits this may introduce within medical education [20]. We believe that restructuring is an important means of engaging and empowering trainees, particularly those that identify as women or are underrepresented in medicine. Social media platforms are an atmosphere where trainees may feel more comfortable using their developing academic voices. However, social media is merely a microcosm of the larger academic medical system; we encourage undergraduate and graduate medical educators to consider how skill sets held by trainees could be better used to train the next generation of physicians alongside the current one.

## Abbreviations

LGBTQ+: lesbian, gay, bisexual, transgender, queer
SMART: specific, measurable, achievable, relevant, and time-bound
WIM: Women in Medicine
